# Water–Fertilizer Synergistic Effects and Resource Optimization for Alfalfa Production: A Central Composite Design and Response Surface Methodology Approach

**DOI:** 10.3390/plants14050731

**Published:** 2025-02-27

**Authors:** Gaiya Mu, Yuanbo Jiang, Haiyan Li, Sinan Wei, Guangping Qi, Yanxia Kang, Minhua Yin, Yanlin Ma, Yayu Wang, Yanbiao Wang, Jinwen Wang

**Affiliations:** College of Water Conservancy and Hydropower Engineering, Gansu Agricultural University, Lanzhou 730070, China; 107332202335@st.gsau.edu.cn (G.M.); 1073323010121@st.gsau.edu.cn (Y.J.); 107332201100@st.gsau.edu.cn (H.L.); 107332202336@st.gsau.edu.cn (S.W.); yanxiakang@gsau.edu.cn (Y.K.); yinmh@gsau.edu.cn (M.Y.); mayl@gsau.edu.cn (Y.M.); wangyy@gsau.edu.cn (Y.W.); 17794437957@163.com (Y.W.); 17899318896@163.com (J.W.)

**Keywords:** desirability function, hydrosustainable crop, response surface methodology, water–nitrogen–phosphorus coupling, water use efficiency, yield

## Abstract

This study posits that strategically optimizing irrigation and fertilization regimes can enhance the productivity and water use efficiency (WUE) of alfalfa (*Medicago sativa* L.), thereby mitigating the constraints imposed by soil impoverishment and water scarcity in forage production systems of arid and semi-arid regions. Conducted over two years, the outdoor pot experiment investigated the effects of water regulation during the branching and bud stages (each at 60–100% θ_0.85_, where θ_0.85_ = 0.85θfc) and different levels of nitrogen and phosphorus fertilization (0–280 kg/ha each) on alfalfa yield and WUE. Using Response Surface Methodology (RSM) with a Central Composite Design (CCD), we modeled the relationships between input variables and key response parameters: total yield, evapotranspiration (ET), and WUE. The response surface models exhibited high reliability, with coefficients of determination *R*^2^, adjusted *R*^2^, predicted *R*^2^, and adequate precision exceeding 0.94, 0.90, 0.86, and 13.6, respectively. Sensitivity analysis indicated that water regulation during critical growth stages, particularly the branching stage, had the most significant impact on yield and ET, while nitrogen and phosphorus fertilization positively influenced WUE. Within the appropriate range of water management, judicious fertilization significantly enhanced alfalfa production performance, although excessive inputs resulted in diminishing returns. This study identified the optimal conditions for sustainable production: branching stage water regulation (82.26–83.12% θ_0.85_) and bud stage water regulation (78.11–88.47% θ_0.85_), along with nitrogen application (110.59–128.88 kg/ha) and phosphorus application (203.86–210 kg/ha). These findings provide practical guidelines for improving the sustainability and efficiency of alfalfa production in resource-limited environments.

## 1. Introduction

Global demand for high-quality forage has surged, driven by the rapid expansion of the livestock industry [1]. Alfalfa (*Medicago sativa* L.), known as the “Queen of Forages”, has gained prominence worldwide due to its exceptional nutritional profile (crude protein content of 15–22%), high biomass yield potential, excellent palatability, and ecological benefits including soil improvement and carbon sequestration [2,3,4]. In China, the conservative estimate of high-quality alfalfa demand has reached 5.6 million tonnes annually, with domestic production and imports each accounting for approximately 50% of the total supply [5]. Within this supply–demand framework, China’s ongoing agricultural restructuring and the progressive implementation of national ecological initiatives have driven the expansion of alfalfa cultivation across the arid and semi-arid regions of Northwest China [6]. However, this expansion has posed unprecedented challenges to water and soil resource management in these regions. As a water-intensive crop, alfalfa’s increasing water demand conflicts with the region’s limited water resources, creating a critical sustainability challenge [7]. The situation is particularly concerning given that the total water supply remains constant while agricultural water requirements continue to rise [8]. Furthermore, due to limited arable land availability, farmers often resort to increasing fertilizer application to boost yields. This practice has led to a series of potential negative effects, including soil nutrient imbalance, water pollution, nitrogen deposition, and greenhouse gas emissions, with nitrous oxide (N_2_O) emissions being particularly prominent [9,10]. Reports indicate that N_2_O emissions from chemical fertilizer application alone account for 88.34% of the total emissions from agricultural lands in China [11]. In response to these challenges, various water management and fertilization optimization strategies have emerged. These approaches aim to achieve multiple objectives: conserving water resources and minimizing environmental impacts, while simultaneously improving crop yields and water use efficiency—key agricultural indicators in sustainable farming systems.

Deficit irrigation (DI) represents an efficient water conservation strategy that intentionally induces moderate water stress to enhance root development and water use efficiency (WUE) without significantly compromising crop yield [12,13]. Renowned for its robust adaptability to drought conditions, alfalfa (*Medicago sativa* L.) stands out as an ideal candidate for deficit irrigation strategies. Extensive research has demonstrated that alfalfa can sustain high yields under deficit irrigation, leveraging a suite of physiological and molecular mechanisms that bolster its drought resistance [14,15]. Studies have pinpointed key genes implicated in phenylpropanoid biosynthesis, starch and sucrose metabolism, and glutathione metabolism, which are instrumental in mediating alfalfa’s response to drought stress [16,17,18,19]. Controlled alternate partial root-zone deficit irrigation (CAPRDI) has been shown to effectively achieve both stable alfalfa production and water conservation [20]. However, this technique can be complex to manage and may not be easily adopted by small-scale farmers. An alternative irrigation approach involves timing-based deficit irrigation, which aligns water management with alfalfa’s phenological water requirements. Studies indicate that a moderate water deficit during a single growth period can effectively maintain soil moisture while achieving high production efficiency. The early-bud stage has been identified as the optimal timing for implementing growth regulation in alfalfa [21]. Nonetheless, the identification of drought-sensitive phenological stages and appropriate deficit levels remains challenging due to variations in geographical location, climatic conditions, soil characteristics, and irrigation methods. These factors significantly influence alfalfa’s response to water stress, with reported yield responses varying from −5% to −40% under similar deficit levels across different regions [22].

With the widespread adoption of fertilization techniques in large-scale alfalfa production, understanding the synergistic effects between water and fertilizers has become crucial for optimizing irrigation and fertilization strategies [22]. While alfalfa yield demonstrates higher sensitivity to water availability compared to nitrogen fertilization, nutrient management remains a critical factor that significantly influences soil water storage and regulation [23]. Fertilizer application can effectively mitigate the adverse effects of drought stress on plant growth and enhance physiological functions [24]. Previous studies have demonstrated that appropriate water–nitrogen ratios can enhance both alfalfa yield and water use efficiency (WUE) [25,26], while water–phosphorus coupling has been shown to promote fine root turnover and dry matter accumulation [27]. However, considerable controversy persists regarding the effects of nitrogen and phosphorus fertilization on alfalfa growth. Some researchers argue that alfalfa can meet its nitrogen requirements through symbiotic nitrogen fixation, eliminating the need for additional nitrogen inputs. This biological process, however, demands substantial phosphorus resources to support root development and drive energy-intensive processes [28]. The divergent findings in previous research may be largely attributed to variations in soil texture and nitrogen application timing. Studies have revealed that rhizobial activity and nodulation stability fluctuate with seasonal changes and soil temperature variations [29,30,31]. Consequently, moderate nitrogen supplementation may benefit legume development, including alfalfa, during periods of weak biological nitrogen fixation, post-harvest recovery, and low soil nitrogen availability [32]. While addressing these controversial aspects, our investigation integrates deficit irrigation with precision fertilization to propose a comprehensive water and nutrient management strategy that simultaneously considers multiple objectives, including crop yield, water consumption, and water use efficiency (WUE). This integrated approach has received limited attention in previous research. While previous studies have primarily focused on dual-factor water–fertilizer coupling, investigations examining the combined effects of multiple factors on *Medicago sativa* L. remain limited, largely due to the complex interactions among multiple water–fertilizer coupling factors [33]. Furthermore, multi-factor, multi-level experiments significantly increase the number of treatments, resulting in higher time and cost investments while intensifying operational challenges.

Given the limitations of traditional research approaches in multi-factor optimization, this study presents an integrated optimization methodology combining Central Composite Design (CCD) and Response Surface Methodology (RSM). This approach offers several advantages, including reduced experimental treatments, multi-response optimization capabilities, clear elucidation of factor interactions, and enhanced prediction accuracy [34,35,36]. While CCD-RSM has been successfully implemented across various disciplines, including chemical engineering [37], pharmaceutical sciences [38], food technology [39], mechanical engineering [40], and metabolomics [41], its application in agricultural water–fertilizer management remains relatively limited. To address this research gap, a two-year pot experiment was conducted incorporating four critical factors: water regulation during the branching stage, water regulation during the bud emergence stage, nitrogen fertilization, and phosphorus fertilization. Through the integrated optimization of water and fertilizer management strategies using CCD-RSM, sustainable and efficient alfalfa production practices will be achieved. The research objectives are as follows: (i) develop multivariate regression models to elucidate the interactive effects of these four factors on alfalfa yield accumulation and water use; (ii) investigate the synergistic advantages of deficit irrigation and balanced N-P fertilization in alfalfa production, addressing the challenges of sustainable grassland agriculture in water- and nutrient-limited regions; and (iii) optimize multi-objective water–fertilizer management strategies using desirability functions to maximize yield, optimize water consumption, and enhance WUE, ultimately providing science-based recommendations for efficient alfalfa production across varying annual conditions in arid and semi-arid regions.

## 2. Results

### 2.1. Overall Evaluation of Water and Fertilizer Regulation

In this study, Response Surface Methodology (RSM) was employed to optimize the water–fertilizer management regime for alfalfa production. Table 1 presents the results of the relevant test parameters based on the Central Composite Design (CCD) model. Prior to model development, preliminary analysis and comprehensive evaluation of key measured parameters, such as yield, water consumption (ET), and water use efficiency (WUE), were conducted to identify factor correlations and underlying mechanisms, thereby establishing a scientific foundation for subsequent modeling and optimization.

**Figure 1:**
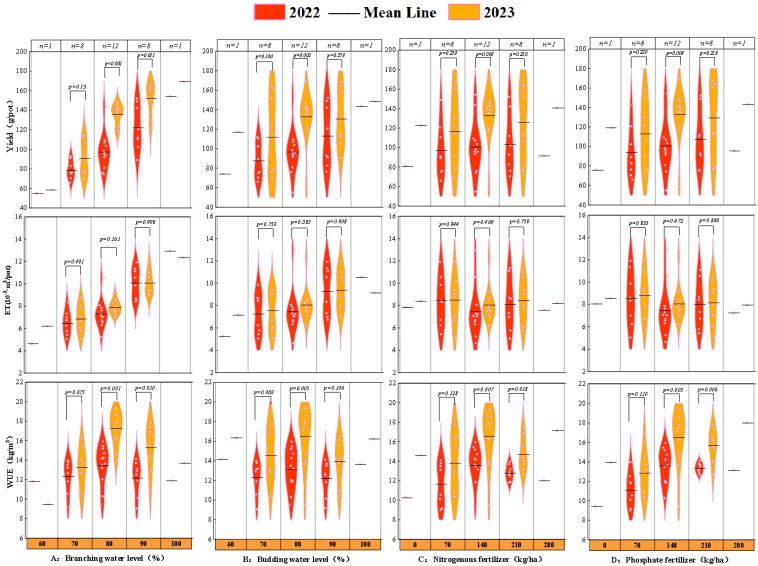
The violin plots of experimental run response variables (yield, water consumption (ET), and water use efficiency (WUE)) versus the independent factors of branching stage water (**A**), budding stage water (**B**), nitrogenous fertilizer (**C**), and phosphate fertilizer (**D**), respectively. In the plot, n denotes the number of replicates corresponding to each specific level of a given factor.

Figure 1 illustrates the violin plots of response variables against four treatment factors. The results indicate that yield increased with the increase in water availability during the branching (bud) stage. Notably, a 90% moisture gradient yielded the widest yield variation range compared to other water levels. Although increased irrigation promoted higher yields, it came at the cost of excessive water consumption and significantly compromised WUE. Consistently observed over the two-year trial, both yield and water consumption rose with increased water input, whereas water use efficiency peaked at an 80% water supply during both the branching and budding stages, subsequently declining as further water input yielded diminishing returns. In terms of fertilization response, both yield and WUE exhibited quadratic responses to nitrogen (phosphorus) fertilization, initially increasing and then declining with higher nutrient inputs, while water consumption reached its minimum at moderate fertilization levels.

This study uncovered significant disparities in the experimental observations between the years 2022 and 2023. Violin plot analyses indicated that under moderate water and fertilizer gradients (with water at 80% or fertilization at 140 kg/ha), both yield and water use efficiency (WUE) demonstrated highly significant differences between the two years (*p* < 0.01), whereas water consumption did not exhibit noteworthy variation (*p* > 0.05). Although 2023 generally exhibited higher yield levels and superior WUE across most treatments compared to 2022, the inter-annual yield difference averaged merely 3.96 g/pot when the water level was maintained at 60% during the branching stage. Notably, WUE under this condition was even lower than that observed in 2022. In contrast, at a water level of 80%, the mean yield difference reached 38.23 g/pot, accompanied by a WUE increase of 3.8 kg/m^3^. These findings suggest that appropriate water deficits and fertilizer application can “harden” the crops, enhancing alfalfa’s productivity in the subsequent year without substantially increasing water consumption. However, severe water stress, particularly during the branching stage, may induce mechanical damage to the root system or cause physiological dysfunction, consequently impacting yield accumulation in the following year, offsetting the intended benefits. Therefore, the optimal water control level should be carefully calibrated to maximize short-term gains without compromising long-term sustainability.

### 2.2. Statistical Analysis of Models

#### 2.2.1. Model Verification

Based on 30 sets of experimental data, this study established regression equations for total yield, water consumption, and water use efficiency of Gansu No. 3 alfalfa during both the first (2022) and second (2023) growing seasons. The significance of various factors and goodness of fit of the regression models were evaluated through analysis of variance (ANOVA). According to the fitting results, unaliased quadratic regression models were used (*p* < 0.01, *R*^2^ > 0.85). The complete models without term reduction, expressed in coded form (Equations (1)–(6)), along with their statistical evaluation metrics, are summarized in Table 2, where (+) and (−) coefficients indicate synergistic and antagonistic effects on response variables, respectively.

All models exhibited high *F*-values with *p* < 0.0001, indicating strong statistical significance. The non-significant Lack of Fit (LF) relative to pure error (*p* > 0.05) confirmed that these models adequately fit the experimental data within their respective regression regions. Therefore, these fitted regression models can be used for statistical analysis of the experimental results. Moreover, the Signal-to-Noise Ratios (APs) of the six models were high, at 22.6089, 29.3266, 13.6607, 25.0582, 25.3256, and 19.1064, respectively, indicating their suitability for prediction.

**Figure 2:**
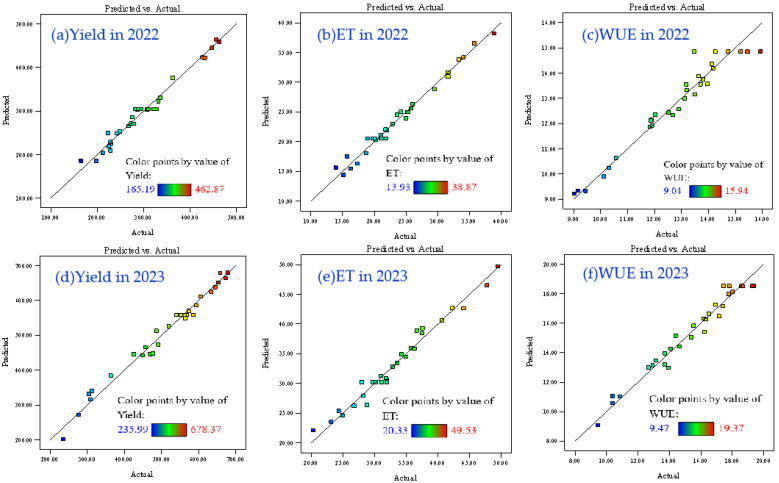
The predicted versus actual values plots of the experimental results: yield in 2022 (**a**) and 2023 (**d**), water consumption (ET) in 2022 (**b**) and 2023 (**e**), and water use efficiency (WUE) in 2022 (**c**) and 2023 (**f**).

The Coefficient of Determination (*R*^2^) exceeded 0.94 for all models, demonstrating a strong correlation between observed and predicted values, which was further validated by the results shown in Figure 2. To account for the potential inflation of *R*^2^ values with increasing predictor variables, adjusted *R*^2^ values were calculated. The adjusted *R*^2^ (*AR*^2^) values (0.9528, 0.9679, 0.9000, 0.9635, 0.9501, and 0.9361) suggested that a high proportion of response variable variation could be attributed to the selected independent variables, with minimal experimental error. The predicted *R*^2^ (*PR*^2^) values aligned well with the adjusted *R*^2^ values (difference < 0.2) and exceeded 0.85, demonstrating robust model generalization capability. These findings confirm that the models effectively explain and estimate experimental parameters while being suitable for design space navigation.

**Figure 3:**
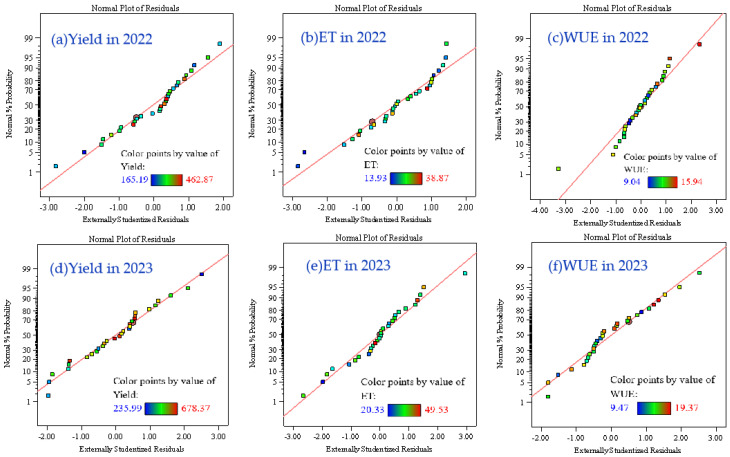
The normal probability plots of the experimental results: yield in 2022 (**a**) and 2023 (**d**), water consumption (ET) in 2022 (**b**) and 2023 (**e**), and water use efficiency (WUE) in 2022 (**c**) and 2023 (**f**).

Model diagnostics were performed using externally studentized residual normal probability plots. As illustrated in Figure 3, the residual points predominantly clustered along the normal distribution line without outliers, confirming the normality assumption and validating model applicability [42].

#### 2.2.2. Analysis of Models

**Figure 4:**
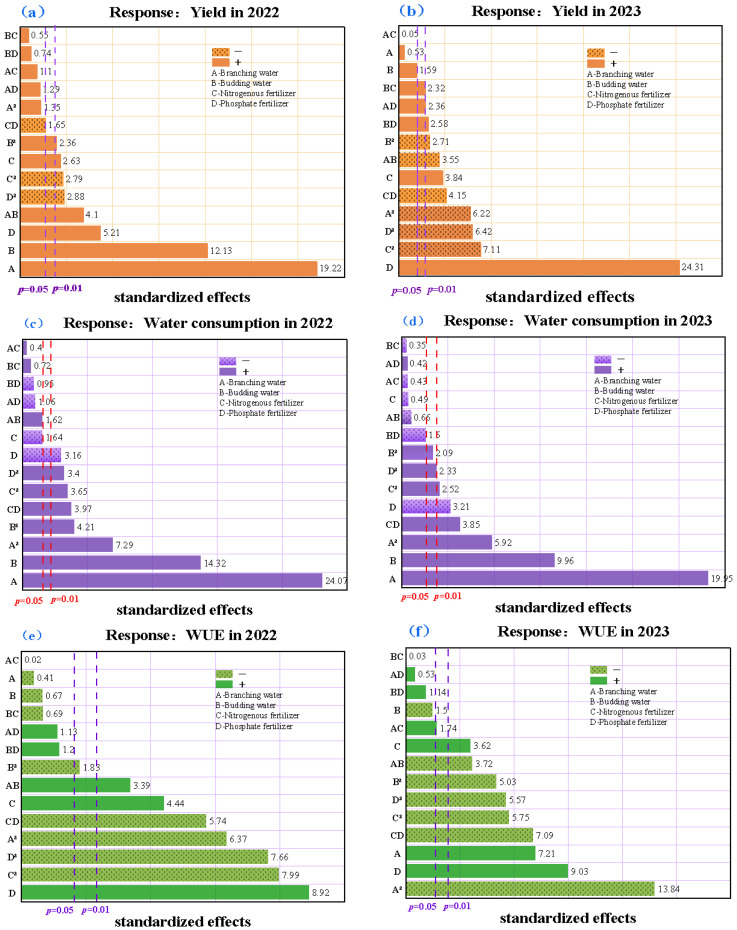
Pareto chart of standardized effects for yield in 2022 (**a**) and 2023 (**b**), water consumption in 2022 (**c**) and 2023 (**d**), and water use efficiency (WUE) in 2022 (**e**) and 2023 (**f**). Here, factor A represents water during the branching stage, factor B represents water during the budding stage, factor C denotes nitrogen fertilization, and factor D signifies phosphorus fertilization. The x-axis represents the coefficient of each parameter in the regression model divided by its standard error. All parameters extending beyond the vertical dotted line at *p* = 0.05 (*p* = 0.01) have significant (highly significant) effects on the response variable.

The predictive models for the three response variables in 2022 demonstrated superior performance compared to those in 2023, with the water consumption model of 2022 exhibiting the highest *R*^2^, *AR*^2^, *PR*^2^, and *AP* indices (Table 2). To evaluate the contribution of significant variables, interaction effects, and quadratic terms to the selected models, Pareto charts were employed; the x-axis represents the standardized effects of each parameter in the regression model (Figure 4). In the 2022 yield regression model, factors A (branching stage water), B (budding stage water), D (phosphorus fertilizer), and the interaction term AB exhibited highly significant effects on yield (*p* < 0.01), while C (nitrogen fertilizer) and quadratic terms A^2^, B^2^, C^2^, and D^2^ showed significant effects (*p* < 0.05). As illustrated in Figure 4a, soil moisture at the branching stage (A) and budding stage (B) had the most substantial impact on the 2022 yield, accounting for 33.14% and 20.91% of the effect, respectively, representing the primary yield-determining factors. The subsequent influential factors, in order of importance, were D, AB, D^2^, C^2^, C, and B^2^, contributing 8.98%, 7.06%, 5.40%, 4.97%, 4.81%, and 4.54% to the model, respectively.

The influence and importance of various factors in the regression models for the same response variable differed between years. The 2023 yield model revealed more pronounced effects of the experimental factors. Specifically, factors A (branching stage moisture), B (budding stage moisture), C (nitrogen fertilizer), D (phosphorus fertilizer), interaction terms AB and CD, and the quadratic term A^2^ all exhibited highly significant effects (*p* < 0.01). Additionally, the interaction term AD and quadratic terms B^2^, C^2^, and D^2^ showed significant impacts on yield (*p* < 0.05). The Pareto chart (Figure 4b) highlighted the positive and significant influences of factors A, B, D, and C and the interaction term AD on the model, with contribution rates of 35.89%, 10.50%, 9.47%, 5.67%, and 6.13%, respectively. Notably, soil moisture at the branching stage remained the most critical factor affecting yield formation. It is worth noting that while the quadratic terms A^2^ and B^2^ showed positive effects in the 2022 yield model, they exhibited negative effects in the 2023 yield model. This shift suggests that excessive soil moisture may inhibit yield formation, emphasizing the importance of optimal water management in alfalfa production.

Analysis of the water consumption model for 2022 revealed that factors A (branching stage water), B (budding stage water), D (phosphorus application), the interaction term CD, and quadratic components A^2^, B^2^, C^2^, and D^2^ all exerted highly significant influences (*p* < 0.01). Among these, factors A and B, along with their quadratic terms A^2^ and B^2^, made the most substantial contributions to the model, with contribution rates of 34.16%, 10.84%, 10.35%, and 5.97%, respectively (Figure 4c). For the 2023 water consumption model, factors A and B, interaction term CD, and quadratic term A^2^ demonstrated highly significant effects (*p* < 0.01), while factor D and quadratic terms C^2^ and D^2^ exhibited significant impacts (*p* < 0.05). As illustrated in Figure 4d, factors A, B, A^2^, and CD exerted the strongest influence on the model, with contribution rates of 37.09%, 18.52%, 11.00%, and 7.19%, respectively.

Water use efficiency (WUE) serves as a crucial metric for evaluating the relationship between crop yield and water consumption, where higher WUE values indicate more efficient water resource utilization. The results revealed that factors C (nitrogen fertilizer), D (phosphorus fertilizer), interaction terms AB and CD, and quadratic terms A^2^, C^2^, and D^2^ had highly significant effects (*p* < 0.01) on WUE in 2022. The contribution rates of significant terms to the model followed the order of D > C^2^ > D^2^ > A^2^ > CD > C > AB (17.68%, 15.83%, 15.18%, 12.62%, 11.38%, 8.80%, and 6.72%). Notably, nitrogen fertilizer (D), phosphorus fertilizer (C), and interaction term AB exhibited positive effects (Figure 4e). In the 2023 WUE model, factors A, C, D, interaction terms AB and CD, and all quadratic terms (A^2^, B^2^, C^2^, and D^2^) showed highly significant influences (*p* < 0.01). Their contribution rates followed the sequence A^2^ > D > A > CD > C^2^ > D^2^ > B^2^ > AB > C (21.04%, 13.72%, 10.96%, 10.77%, 8.75%, 8.46%, 7.64%, 5.66%, and 5.51%), with single factors A, D, and C demonstrating positive effects (Figure 4f).

### 2.3. Single and Interactive Effects of Process Parameters

#### 2.3.1. Effects on Hay Yield

**Figure 5:**
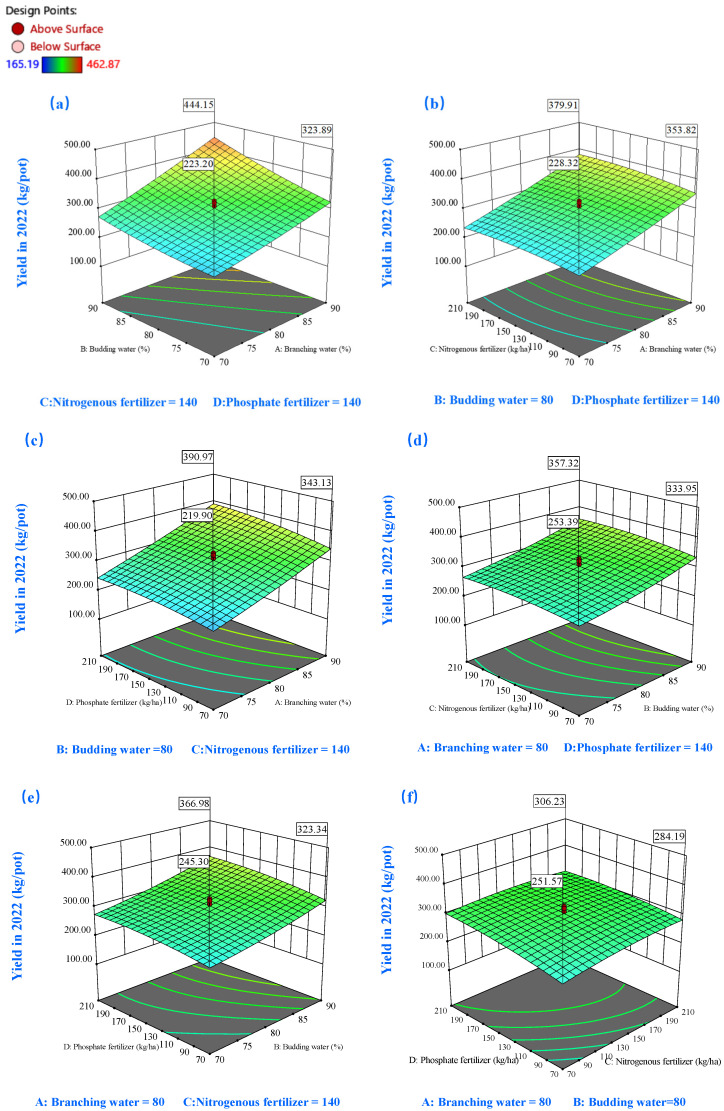
The 3D response surface plots of the interaction of AB (**a**), AC (**b**), AD (**c**), BC (**d**), BD (**e**), and CD (**f**) on yield in 2022. Here, factor A represents water during the branching stage, factor B represents water during the budding stage, factor C denotes nitrogen fertilization, and factor D signifies phosphorus fertilization.

**Figure 6:**
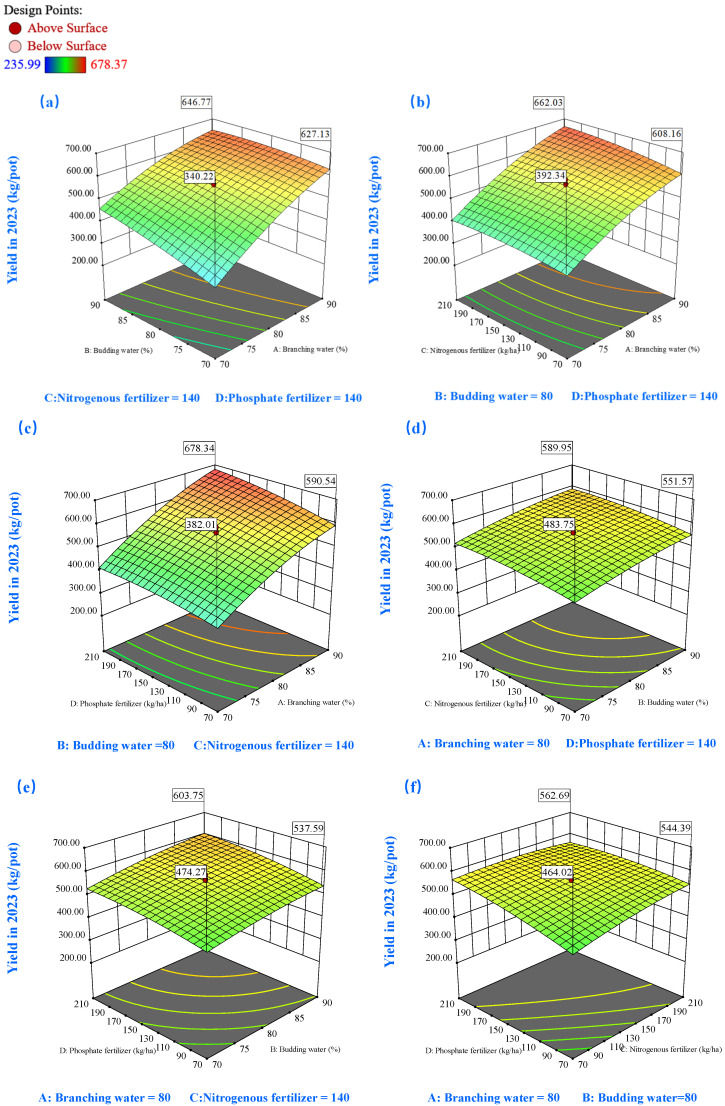
The 3D response surface plots of the interaction of AB (**a**), AC (**b**), AD (**c**), BC (**d**), BD (**e**), and CD (**f**) on yield in 2023. Here, factor A represents water during the branching stage, factor B represents water during the budding stage, factor C denotes nitrogen fertilization, and factor D signifies phosphorus fertilization.

**Figure 7:**
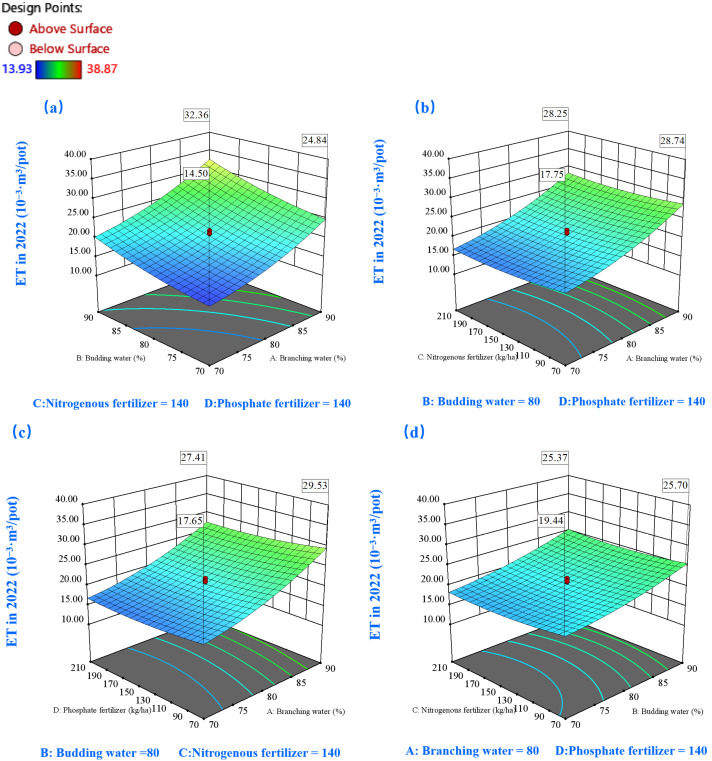

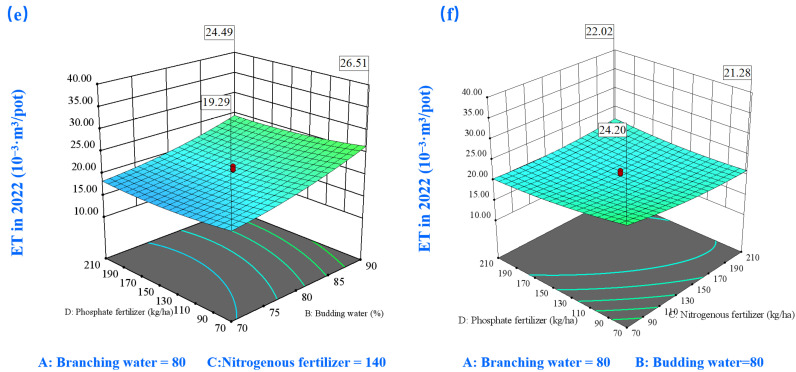
The 3D response surface plots of the interaction of AB (**a**), AC (**b**), AD (**c**), BC (**d**), BD (**e**), and CD (**f**) on water consumption (ET) in 2022. Here, factor A represents water during the branching stage, factor B represents water during the budding stage, factor C denotes nitrogen fertilization, and factor D signifies phosphorus fertilization.

**Figure 8:**
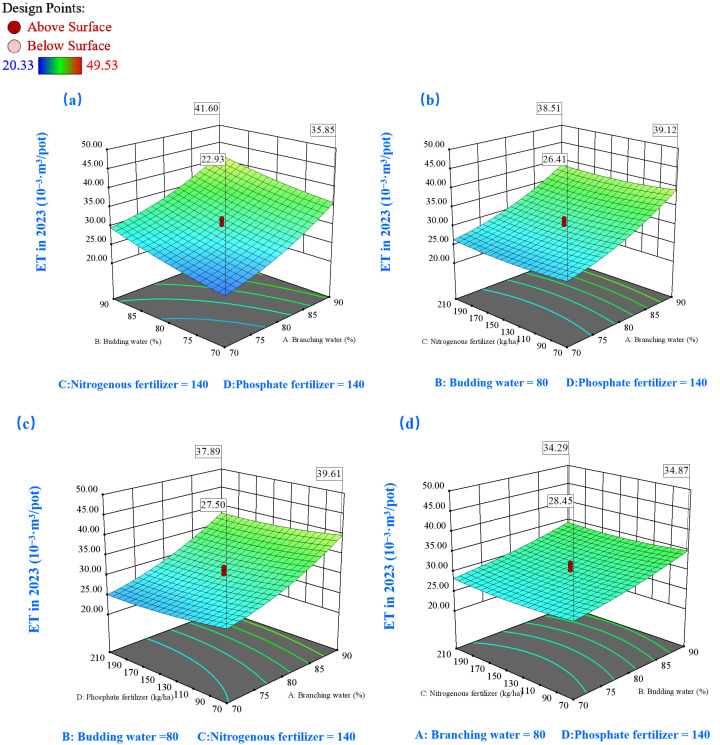

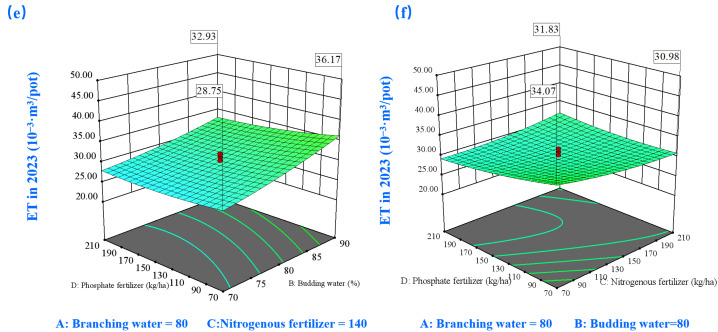
The 3D response surface plots of the interaction of AB (**a**), AC (**b**), AD (**c**), BC (**d**), BD (**e**), and CD (**f**) on water consumption (ET) in 2023. Here, factor A represents water during the branching stage, factor B represents water during the budding stage, factor C denotes nitrogen fertilization, and factor D signifies phosphorus fertilization.

**Figure 9:**
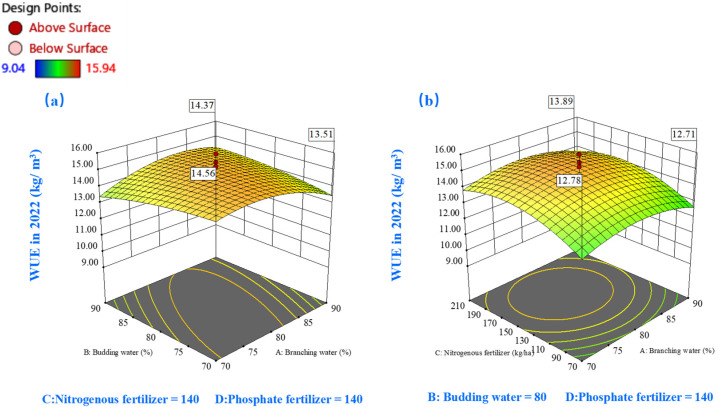

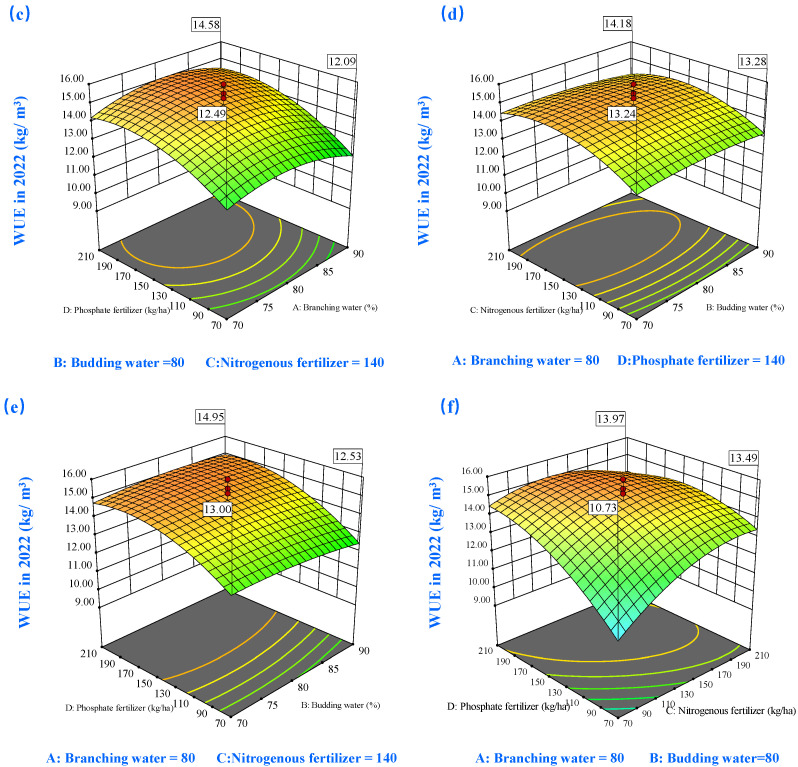
The 3D response surface plots of the interaction of AB (**a**), AC (**b**), AD (**c**), BC (**d**), BD (**e**), and CD (**f**) on water use efficiency (WUE) in 2022. Here, factor A represents water during the branching stage, factor B represents water during the budding stage, factor C denotes nitrogen fertilization, and factor D signifies phosphorus fertilization.

**Figure 10:**
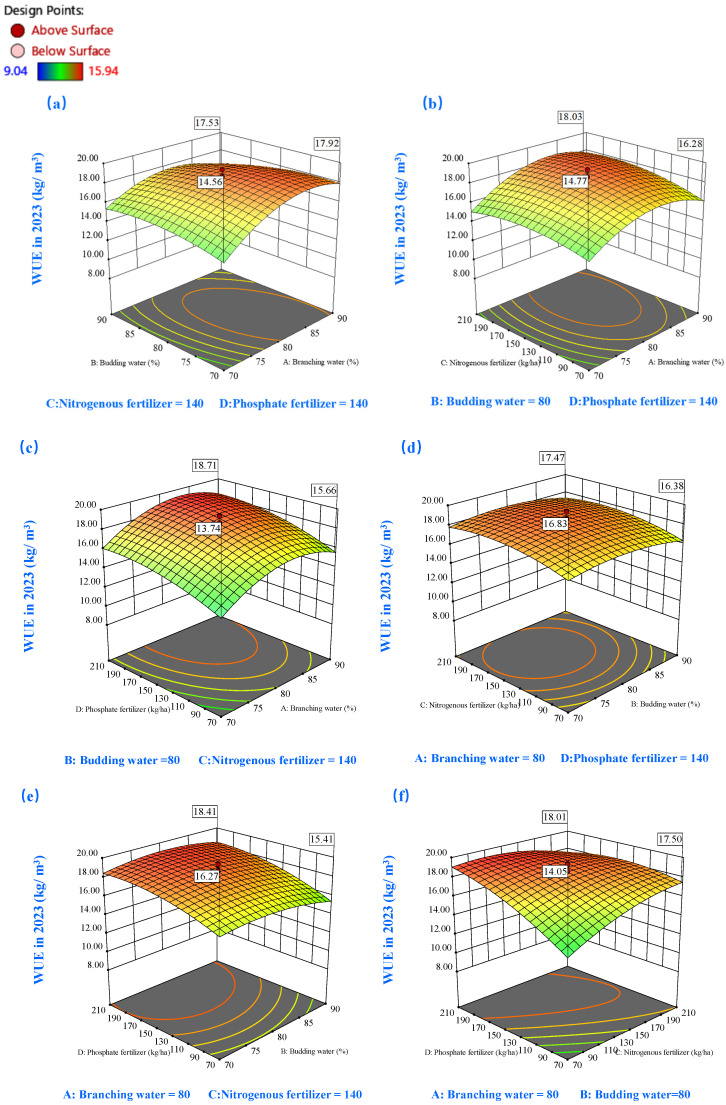
The 3D response surface plots of the interaction of AB (**a**), AC (**b**), AD (**c**), BC (**d**), BD (**e**), and CD (**f**) on water use efficiency (WUE) in 2023. Here, factor A represents water during the branching stage, factor B represents water during the budding stage, factor C denotes nitrogen fertilization, and factor D signifies phosphorus fertilization.

Three-dimensional response surfaces were generated to elucidate the relationships between key variables and their optimal values, with two factors fixed at their median levels (Figure 5, Figure 6, Figure 7, Figure 8, Figure 9 and Figure 10). Analysis revealed that that biomass production exhibited a marked upward trend with simultaneous elevation of soil moisture content during both the branching and budding stages, as evidenced by the steep gradient of the response surface (Figure 5a). Under controlled soil moisture conditions, yield response to nitrogen (C) and phosphorus (D) fertilizers exhibited a characteristic parabolic pattern marked by an initial increase followed by a decrease (Figure 5b–e). This trend suggests that excessive fertilizer input may be detrimental to alfalfa hay yield accumulation, corroborated by the negative effects of D^2^ and C^2^ terms in the model (Figure 4a). Furthermore, higher soil moisture levels enhanced fertilizer efficacy. As shown in Figure 5d, when soil moisture during branching was maintained at 70%, increasing phosphorus fertilizer from 70 to 210 kg/ha resulted in a yield range of 218.19–244.12 g/pot. In contrast, under 90% moisture conditions, the yield range expanded to 343.05–390.53 g/pot.

The second-year response surface analysis (Figure 6) revealed patterns consistent with first-year observations, albeit with notable distinctions. In the second year, alfalfa yield showed heightened sensitivity to soil moisture and fertilizers, evidenced by more pronounced heat effects in the response surface plots. Under identical soil moisture conditions (90% water level during branching), P application induced biomass variations ranging from 591.13 to 679.0 g/pot, marking a substantial improvement over the previous year’s range of 333.7 to 357.38 g/pot (Figure 6c). The interaction between N and P fertilization displayed complex dynamics (Figure 6f). At lower N applications (70–140 kg/ha), P fertilization demonstrated enhanced efficiency, resulting in significant biomass improvements. However, N applications exceeding 140 kg/hm^2^ led to diminishing returns in P utilization efficiency, eventually showing a slight decline beyond certain P thresholds. The interaction term (CD) exhibited antagonistic characteristics, contributing a significant 4.80% to the model variance (Figure 4b).

#### 2.3.2. Effects on Water Consumption (ET)

Response surface analysis of water consumption patterns across 2022–2023 (Figure 7 and Figure 8) revealed consistent temporal trends in relation to experimental variables. A notable correlation emerged between soil moisture elevation and increased water consumption, as evidenced in both annual datasets (Figure 7a and Figure 8a). Our investigation uncovered an intriguing phenomenon under severe moisture stress conditions (70% water level during branching): supplementary nutrient application, particularly N or P fertilization, demonstrated potential for moderating alfalfa’s water uptake (Figure 7b,c and Figure 8b,c). This observation carries substantial implications for moisture conservation strategies in moisture-limited environments. However, the relationship exhibited a threshold effect: beyond certain nutrient application levels, water consumption patterns showed an upward trajectory.

The nutrient interaction analysis for 2022 yielded compelling insights: water consumption efficiency peaked under various nutrient combinations, including scenarios with asymmetric N-P ratios (high N-low P or vice versa) or balanced moderate applications (Figure 7f). These findings underscore the criticality of strategic nutrient management for the simultaneous optimization of growth parameters and water resource efficiency. The 2023 response surface analysis further refined our understanding, indicating optimal water–nutrient dynamics at P application rates of 155–210 kg/ha coupled with N rates of 70–150 kg/ha (Figure 8f).

#### 2.3.3. Effects on Water Use Efficiency (WUE)

The WUE response surfaces for both study years (Figure 9 and Figure 10) exhibited a characteristic parabolic relationship with soil moisture content. In 2022, peak WUE values (exceeding 14.7 kg/m^3^) were observed within a specific branching-stage moisture window (74–78%), while bud-stage moisture requirements remained flexible (70–90%) (Figure 9a). In the subsequent year, enhanced WUE performance (>18 kg/m^3^) was demonstrated at slightly elevated branching-stage moisture levels (80–89%), with bud-stage requirements ranging from 70% to 88% (Figure 10a). The moisture–nutrient interaction analysis highlighted the effectiveness of intermediate moisture conditions (80%) combined with enhanced nutrient availability (>150 kg/ha) in maximizing WUE outcomes (Figure 9c,e and Figure 10c,e).

Temporal analysis revealed subtle yet significant variations in nutrient response patterns. The 2022 dataset (Figure 9f) demonstrated a non-linear WUE response to nitrogen application, with optimal performance observed under moderate-to-high P (150–210 kg/ha) and low-to-moderate N (100–160 kg/ha) conditions, yielding WUE values above 15 kg/m^3^. Figure 10f illuminated distinctive response patterns: WUE exhibited heightened sensitivity to nutrient adjustments under lower baseline conditions while demonstrating relative stability at elevated nutrient levels. The 2023 data specifically indicated superior WUE performance (>19 kg/m^3^) under conditions favoring high P (>176 kg/ha) combined with conservative N application (70–146 kg/ha).

### 2.4. Optimization of Water and Fertilizer Management Strategies

The complexity of multiple Response Surface Methodology (RSM) factors necessitates a sophisticated optimization approach beyond direct surface analysis. To address this challenge, we implemented the Desirability Function Approach (DFA), an advanced mathematical optimization technique, to establish precise water–fertilizer management protocols for alfalfa cultivation. The overall model desirability was determined through numerical optimization, with desirability values ranging from 0 (below limit) to 1 (target achieved). Specific optimization criteria were established for each independent variable and response value, as summarized in Table 3.

**Figure 11:**
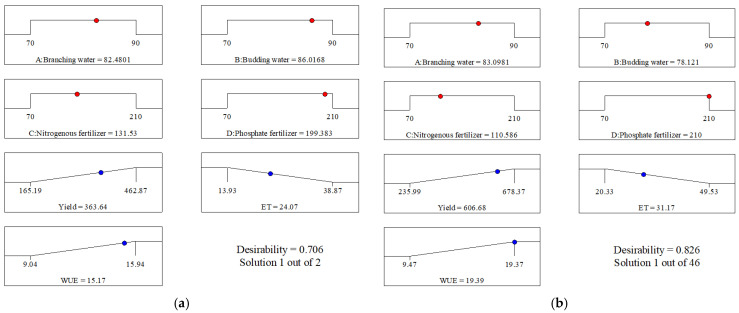
Desirability ramp for numerical optimization of goals in 2022 (**a**) and 2023 (**b**). Optimal factor settings are shown with red points and optimal response prediction values are displayed in blue. Here, ET denotes water consumption and WUE refers to water use efficiency.

When minimizing water consumption was prioritized as a response variable, yield was inevitably constrained. Sensitivity analysis revealed (Figure 4) that water availability consistently demonstrated a significant contribution to yield across both growing seasons, with a total contribution rate of 54.05% in 2022 and 46.39% in 2023. However, water use efficiency (WUE) did not exhibit synchronized changes with yield and water consumption. As water supply increased, both yield and water consumption concurrently rose, while WUE peaked at moderate water levels before declining (Figure 9a and Figure 10a). In order to achieve optimal yield outcomes while considering these relationships, we assigned importance weights of 4 to both yield and WUE, and 3 to water consumption (Table 3), resulting in an optimized water–fertilizer combination scheme. Figure 11 illustrates the desirability slopes for numerical optimization of the target values, and the response desirability for each variable is listed in Table 3.

The optimization algorithm generated distinct management strategies for each growing season. For 2022, the optimal configuration emerged with precise moisture control during critical growth stages: 83.72% at branching and 88.47% at budding, complemented by calibrated nutrient applications of 128.88 kg/ha nitrogen and 203.86 kg/ha phosphorus. This protocol achieved a desirability coefficient of 0.721, yielding 389.97 g/pot with water consumption of 26.0 × 10^−3^ m^3^/pot and WUE of 15.15 kg/m^3^ (Figure 11a). Comparative analysis against conventional management practices (A = 100%, B = 80%, C = 140 kg/ha, and D = 140 kg/ha) demonstrated remarkable resource efficiency: 31.58% reduction in water inputs (conservation of 12 × 10^−3^ m^3^/pot) and 27.20% enhancement in WUE, while maintaining acceptable yield reduction of 15.75%.

The 2023 optimization protocol demonstrated enhanced efficiency, achieving a superior desirability coefficient of 0.830. The refined parameter set (branching moisture: 83.12%, budding moisture: 78.11%, nitrogen: 110.59 kg/ha, and phosphorus: 210 kg/ha) generated exceptional results: yield of 606.8 g/pot, water consumption of 31.17 × 10^−3^ m^3^/pot, and WUE of 19.37 kg/m^3^ (Figure 11b). This advanced management strategy achieved remarkable resource conservation metrics: 36.83% water savings (reduction of 18.36 × 10^−3^ m^3^/pot) and 29.27% WUE enhancement while minimizing yield impact to 13.36%. These results underscore the potential for significant agricultural sustainability improvements through precision water–fertilizer management.

## 3. Discussion

In this study, we investigated the effects of water regulation during the branching and bud-forming stages, along with nitrogen and phosphorus fertilization, on alfalfa production. Using a second-order regression model based on the general rotatable combination design principle, we established quadratic regression equations to analyze yield, water consumption, and water use efficiency under coupled water–fertilizer effects. This approach allowed us to examine the individual and interactive impacts of these factors on alfalfa’s production characteristics and optimize water–fertilizer management strategies under multi-objective conditions.

### 3.1. Impact of Water and Fertilizer Regulation on Alfalfa Yield

Our findings from the 2022 and 2023 experiments reveal that the factors influencing alfalfa yield follow the following order: water regulation during the branching stage > water regulation during the bud-forming stage > phosphorus fertilization > nitrogen fertilization. This hierarchy aligns with the results reported by Tian et al. [43]. Regarding water management during the growth stages, our results corroborate the findings of Tong et al. [44], who demonstrated through a two-year field study that water deficit during the branching stage significantly affects alfalfa yield, confirming this period as critical for water requirements. Similarly, Liu et al. [21] identified the branching stage as the most drought-sensitive period for alfalfa, with optimal effects of water stress application during the bud-forming stage, which is consistent with our observations. These findings underscore the distinct response mechanisms of alfalfa to environmental stresses at different growth stages. During the branching stage, plants are in a rapid growth state with active root development and branch growth, making them more susceptible to water shortages. In contrast, by the bud-forming stage, plants have established a physiological foundation, enhancing their adaptability to water variations.

Concerning the impact of fertilizers on yield, the correlation between growth, biomass, and exogenous nutrients has been extensively studied in forage crops. Wu et al. [45], in a five-year localized fertilization experiment, observed that alfalfa total yield initially increased and then decreased with rising fertilization levels, consistent with the law of diminishing returns observed in our study. However, some studies have reported an insignificant yield-increasing effect with nitrogen fertilization in legumes, possibly due to inherently high soil nitrogen content (1.0 g/kg) at experimental sites [46,47]. Based on our yield model, we postulate that alfalfa exhibits a threshold for nutrient uptake. Below this threshold, fertilizers promote growth and development, whereas exceeding the maximum absorption capacity negatively impacts growth and reduces forage yield. Additionally, previous studies have reported that nitrogen and phosphorus fertilization, particularly the latter, significantly enhanced alfalfa hay yield [45,48], with phosphorus exerting a greater promotional effect than nitrogen [49], aligning with our findings. The underlying mechanism may be related to nitrogen addition increasing plant phosphorus demand. Alfalfa acquires phosphorus through rhizosphere acidification and root–mycorrhizal interactions, a process that increases root carbohydrate consumption, potentially limiting nodule growth and function. Conversely, phosphorus fertilization significantly modulates the effects of nitrogen addition, which is becoming increasingly crucial for alfalfa biomass and nodule regulation [50]. Notably, our study observed that alfalfa yield in 2023 showed heightened sensitivity to nitrogen fertilization compared to 2022. This phenomenon can be attributed to gradual soil nutrient depletion, meteorological variations, and differences in alfalfa growth characteristics across years. This observation underscores the necessity of considering soil conditions and climate change effects across different years in future fertilization management to optimize water–fertilizer allocation strategies for sustainable alfalfa production.

### 3.2. Effects of Water and Fertilizer Management on Water Consumption and Water Use Efficiency of Alfalfa

Water consumption management is crucial for alfalfa production, particularly in arid and semi-arid regions where irrigation efficiency optimization is paramount for water conservation. Water consumption and water use efficiency (WUE) serve as fundamental indicators for evaluating irrigation management effectiveness [51]. Our two-season investigation revealed a positive correlation between soil moisture content and water consumption in alfalfa. Under identical soil moisture conditions, fertilization significantly influenced water consumption patterns, with the most pronounced effects observed in the nitrogen–phosphorus interaction. Specifically, water consumption exhibited a U-shaped response to increasing fertilizer applications, aligning with findings reported by Xue et al. [23]. Fertilization acts as a sensitive factor in water consumption by modulating soil water storage and utilization dynamics. Optimal fertilization practices substantially enhance soil water retention capacity and water use efficiency [52,53]. Studies by Thompson [52] and Agami [54] have demonstrated that appropriate fertilization can effectively mitigate the adverse effects of drought stress. While our pot experiments recorded slightly higher water consumption compared to field studies, this disparity may be attributed to the confined root system in potted plants, which limits their ability to access deep soil moisture reserves. The relatively short irrigation intervals (typically 3-day cycles) in pot experiments likely contributed to elevated water consumption. Nevertheless, pot experiments offer the advantage of precise isolation of irrigation treatment effects, enabling a more accurate assessment of how different water deficit levels impact alfalfa growth.

Water regulation factors (A^2^ and B^2^) and nutrient factors, specifically nitrogen (C) and phosphorus (D), demonstrated significant effects on water use efficiency (WUE). The relationship between WUE and soil moisture content followed a quadratic pattern, reaching optimal values at approximately 80% soil moisture content during both growth stages. This observation corroborates the findings of Wang et al. [55] and Liu et al. [56], indicating superior WUE under moderate water stress compared to well-watered conditions. Similarly, WUE exhibited a parabolic response to both nitrogen and phosphorus applications, consistent with observations by Xue et al. [23]. From a thermodynamic perspective, the mechanism by which fertilization improves plant water use efficiency can be attributed to increasing the water thermodynamic function between soil and plants: the gradient of partial molar free energy. This mechanism not only augments the plant’s water absorption capacity but also equilibrates the differences in the water-free energy gradient between soil and plants caused by different soil moisture contents [57].

### 3.3. Multi-Objective Optimization of Alfalfa Production Performance Based on Desirability Function

In contemporary agricultural practices, single-objective evaluation often fails to simultaneously address both resource conservation and production efficiency. Previous research has demonstrated that crop yield optimization and water use efficiency (WUE) do not always exhibit a synchronous relationship [58,59,60]. Our findings also demonstrate a nonlinear relationship between yield and WUE, as yield increases with water supply while changes in WUE follow a downward-opening quadratic curve. This finding underscores the challenge of achieving optimal resource utilization while maintaining high productivity. In arid and semi-arid regions, where sustainable development and effective water resource management have become increasingly critical, there is a pressing need to achieve high crop yields while maintaining minimal water consumption. To address these challenges, we proposed a multi-objective optimization approach using a water–fertilizer coupling model. The Response Surface Methodology (RSM) combined with desirability functions was employed to transform multiple conflicting objectives into a single optimization problem [61]. This methodology has been validated as an effective tool by several researchers, including Divya [62] and Moghaddam [63]. Our study focused on three primary optimization objectives: maximizing crop yield, minimizing water consumption, and maximizing WUE. Systematic optimization was conducted for water–fertilizer application schemes in both the 2022 and 2023 growing seasons. The results demonstrated that optimized combinations achieved high satisfaction levels within acceptable yield reduction ranges. The comprehensive evaluation indicated that under appropriate water–fertilizer conditions, regulated deficit irrigation (RDI) not only enhanced alfalfa’s WUE and hay yield while effectively reducing water consumption but also positively influenced production performance and water utilization in the second growing season. These findings have significant implications for sustainable alfalfa production in arid and semi-arid regions.

The effectiveness of CCD-RSM in studying water–fertilizer coupling effects on alfalfa was validated through model assessment diagnostics and comparative analysis with previous research. However, it is important to acknowledge that water–fertilizer coupling exhibits temporal and spatial heterogeneity, with research outcomes varying across different geographical regions and soil textures. Therefore, practical applications must be adapted to local conditions. Furthermore, alfalfa differs significantly from traditional cereal crops in growth characteristics, production performance, and resource requirements. Long-term field experiments are needed in the future to identify additional potential influencing factors and develop more refined alfalfa production management models integrated with crop modeling systems. This will provide robust theoretical foundations and data support for smart information-based agriculture.

## 4. Materials and Methods

### 4.1. Study Site and Experimental Materials

**Figure 12:**
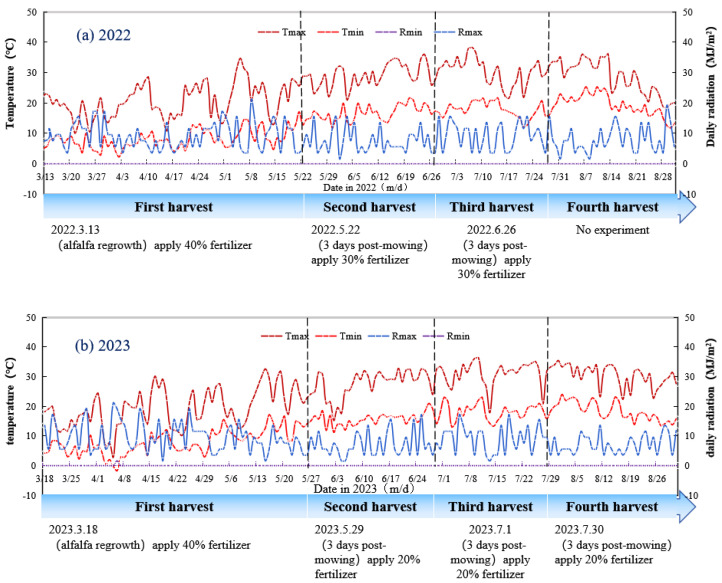
Environmental factors and timeline of the alfalfa growth periods in 2022 (**a**) and 2023 (**b**). Tmax, maximum temperature; Tmin, minimum temperature; Rmax, maximum radiation; and Rmin, minimum radiation.

This study was conducted at the Grassland Training Base of Gansu Agricultural University (103°40′ E, 36°03′ N) during the periods of April to August 2022 and April to September 2023. The experimental site is characterized by a temperate semi-arid continental climate, with an average elevation of 1525 m above sea level. The area experiences a mean annual temperature of 10.3 °C, a frost-free period of 180 days, and annual precipitation ranging from 400 to 600 mm, predominantly concentrated between July and September. The average annual evaporation is 1410 mm, with 2100 to 2600 h of annual sunshine. Phenological stages, temperature, and solar radiation data for the growing seasons of 2022 and 2023 are presented in Figure 12.

The experiment was conducted using pot cultivation. The pots were made of a new type of resin, with a mouth diameter of 25 cm and a height of 30 cm. Each pot was filled with 12.5 kg of mixed soil, which was prepared in a 5:1 ratio of the original soil (loess) from the experimental site and a commercial seedling substrate (Xinxian Luyuan Seedling Substrate, purchased from Xinxian Luyuan Seedling Substrate Co., Ltd., Liaocheng, China). The basic physicochemical properties of the reconstituted soil (0–30 cm) are detailed in Table 4. For the quantification of organic matter, the dichromate oxidation technique was employed, utilizing a 0.8 mol/L solution of potassium dichromate (K_2_Cr_2_O_7_) alongside 98% concentrated sulfuric acid (H_2_SO_4_). The measurement of total nitrogen was achieved via the Kjeldahl digestion process, where soil samples underwent digestion with concentrated sulfuric acid, followed by the distillation of released ammonia and subsequent titration with boric acid. The assessment of available nitrogen was conducted using the alkaline diffusion method, incorporating a 1 M sodium hydroxide (NaOH) solution. Available phosphorus was determined by the molybdenum blue method after extraction with a 0.5 M sodium bicarbonate (NaHCO_3_) solution. Flame photometry was used to analyze available potassium following extraction with 1 M acetic acid. Each chemical analysis was performed in triplicate to ensure the precision and reliability of the results [64].

Before sowing on 8 April 2021, the experimental site was plowed, leveled, and cleared of weeds. Each pot was sown with 40 seeds of alfalfa. The cultivar used was “Gannong No. 3” purple-flowered alfalfa (*Medicago sativa* cv. Gannong No. 3), which is widely cultivated in the arid and semi-arid regions of Northwest China due to its superior comprehensive performance [65,66]. The seeds were provided by the College of Grassland Science, Gansu Agricultural University. When the seedlings reached the three-leaf stage, manual thinning was performed, retaining 30 healthy and uniformly growing alfalfa seedlings per pot. No experimental treatments were applied in the establishment year, and all pots received the same amount of water and fertilizer, which were applied after thinning. The fertilizers used included urea (N ≥ 46.6%) as the nitrogen source, calcium superphosphate (P_2_O_5_ ≥ 16%) as the phosphorus source, and potassium sulfate (K_2_O ≥ 45%) as the potassium source. In addition, daily watering and pest and disease control measures were implemented to ensure the healthy growth of alfalfa. During rainy days, a rain shelter was used to cover the pots, while other field management practices were consistent with local practices.

### 4.2. Research Methods

**Figure 13:**
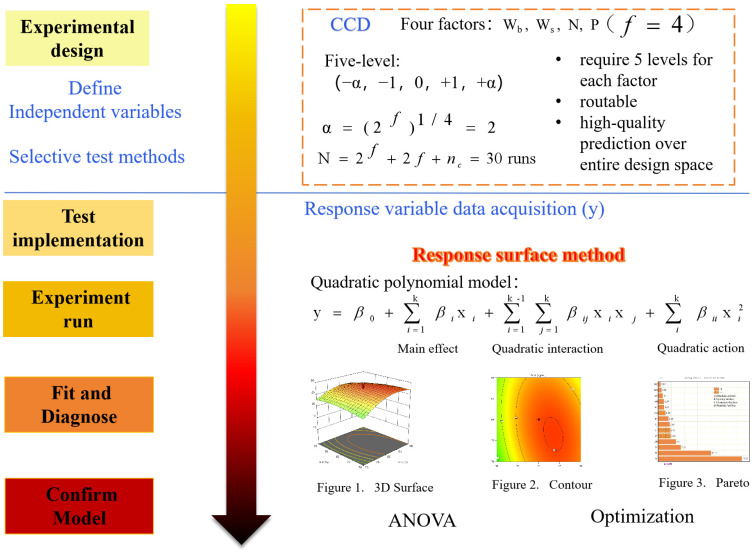
CCD-RSM optimization diagram based on experimental design.

The Response Surface Methodology (RSM) with Central Composite Design (CCD) was employed for experimental design, modeling, and data analysis. The optimization procedure consisted of the following steps [36]: (1) identifying the independent variables closely related to the response variables and their corresponding ranges based on preliminary research [67]; (2) designing the experiments using the CCD module in Design-Expert software (Trial version 13, Stat-Ease Inc., Minneapolis, MN, USA); conducting the experiments and measuring the relevant data (i.e., the primary response variables, including yield, water consumption (ET), and water use efficiency (WUE)) according to the experimental design table; (4) establishing quadratic polynomial equations between the experimental data response variables and the corresponding combinations of independent variables; the basic form of the predictive quadratic polynomial equation used to estimate the response (*y*) based on the independent variables (*x_i_*) is given in Equation (7) [36]; (5) evaluating the accuracy of the constructed models and the significance of the model regression coefficients using analysis of variance (ANOVA); and (6) searching for the optimal combinations of independent variables and the corresponding extreme values of the response variables using RSM or desirability function methods. Figure 13 provides a flowchart illustrating the optimization of a response variable following the above steps:

(7) $$y=\beta_{0}+\sum_{i=1}^{k}\beta_{i}x_{i}+\sum_{i=1}^{k}\beta_{ii}x_{ii}^{2}+\sum_{1\leq i\leq j}^{k}\beta_{ij}x_{i}x_{j}+\varepsilon$$

where

- *y* represents the predicted response variable.
- *β*_0_ is the constant term.
- *k* denotes the total number of independent variables.
- *β_i_* represents the linear coefficient for the *i*-th independent variable.
- *x_i_* and *x_j_* represent the *i*-th and *j*-th independent variables, respectively.
- *β_ij_* is the interaction term coefficient between *x_i_* and *x_j_*.
- *β_ii_* indicates the quadratic coefficient for the *i*-th independent variable.
- *ε* represents the residual of the model, which is the difference between the actual observed values and the values predicted by the model.

#### 4.2.1. Experimental Design

A rotatable Central Composite Design (CCD) was implemented with four independent variables: water regulation during the branching stage (W1), water regulation during the budding stage (W2), nitrogen application rate (N), and phosphorus application rate (P). The total number of experiments was calculated using the formula $N=2^{f}+2f+n_{c}$ [36], where *f* represents the number of factors (*f* = 4) and *n_c_* denotes the number of center point replicates (*n_c_* = 6) for estimating experimental error [68]. The experimental design matrix comprising 30 treatments was generated using the CCD module in Design-Expert software (Trial version 13, Stat-Ease Inc., Minneapolis, MN, USA). Each treatment was replicated five times, resulting in a total of 150 experimental pots. Additionally, the models were established based on the coded values of the independent variables rather than their actual values. The coded values (*x_i_*) of each independent variable were calculated using the following formula [69]:

(8) $$x_{i}=\frac{X_{i}-X_{0}}{\Delta X}$$

where

- *x_i_* is the coded value of the independent variable.
- *X_i_* is the actual value of the independent variable.
- *X*_0_ is the actual value of the independent variable at the center point.
- Δ*X* is the step change value.

The coded factor levels were set as (−*α*, −1, 0, +1, +*α*), where *α* was calculated using the formula $\alpha ={\left(2^{f}\right)}^{1/4}=2$. Thus, the coded factor levels were (−2, −1, 0, +1, +2). The factor coding levels and ranges are detailed in Table 5, while the treatment matrix generated from the Central Composite Design (CCD) is presented in Table 6.

From the branching period in 2022, a dynamic irrigation strategy was implemented to gradually adjust the soil water content in each pot to meet experimental requirements. Soil volumetric water content (VWC) in the 0–30 cm soil profile was monitored using a portable Time Domain Reflectometry device (TRIME-PICO-IPH, IMKO, Ettlingen, Baden-Württemberg, Germany) at three-day intervals, with additional measurements during high-temperature periods. Supplemental irrigation was applied when soil moisture dropped below the predetermined threshold, which was set at 85% of field water capacity (FWC, used as the reference water content throughout this study). To ensure measurement accuracy, TDR readings were validated every seven days using the gravimetric method through oven-drying. The fertilization protocol was designed based on the annual nutrient requirements and the distinct characteristics of nutrient mobility and uptake patterns. Phosphorus and potassium fertilizers were applied as a single dose during the spring green-up period, given phosphorus’s limited mobility in soil and alfalfa’s rapid potassium uptake capacity [70]. Nitrogen application was strategically distributed throughout the growing period, accounting for nutrient uptake patterns and utilization efficiency at different growth stages [71].

The experimental design underwent adaptive modification based on observed growth patterns. Initially, following previous field trials [72], the experiment was structured for three harvests in 2022, with nitrogen applied in a 4:3:3 ratio across the cuts. However, pot cultivation conditions appeared to accelerate alfalfa’s developmental cycle, enabling four harvests annually while maintaining adequate winter hardiness. Consequently, the experimental design was adjusted in the second year to accommodate four harvests, with nitrogen distribution modified to a 4:2:2:2 ratio. Fertilizer applications were conducted by dissolving calculated nutrient quantities in water and applying the solution to each pot 3–5 days after the spring green-up period and subsequent harvests (Figure 12).

#### 4.2.2. Data Collection

(1) Hay Yield

To balance hay yield and nutritional value while ensuring the regrowth of subsequent alfalfa crops, each pot was harvested at the early flowering stage, leaving a stubble height of 5 cm. The harvested biomass was immediately weighed to determine fresh weight and then subjected to a two-step drying process. Samples were first exposed to 105 °C for 30 min to deactivate enzymes, followed by 75 °C for 48 h to achieve constant dry weight. After cooling, the dry weight was recorded as the dry matter yield per pot.

(2) Water Consumption

The water consumption of alfalfa was calculated using the water balance equation [21]:

(9) $$\mathrm{ET}=P+I+\Delta W+E_{P}\;-D$$

where

- *ET* is the total water consumption (mm).
- *I* denotes the volume of irrigation water applied (mm).
- Δ*W* is the change in soil water storage (mm).
- *P*, *E_P_*, and *D* are effective precipitation, groundwater recharge, and deep percolation, respectively, all of which were negligible in this study.

Soil water storage was calculated as follows:

(10) $$W=\frac{h\cdot \theta_{v}}{10}$$

where

- *W* is the soil water storage (mm).
- *h* is the soil layer depth (cm).
- *θ_v_* is the soil volumetric water content (%).

(3) Water Use Efficiency

Water use efficiency (WUE) was calculated as follows [23]:

(11) $$\mathrm{WUE}=\frac{Y}{\mathrm{ET}}$$

where

- *WUE* is the water use efficiency (kg/m^3^).
- *Y* is the total yield of alfalfa (kg/ha).
- *ET* is the total water consumption of alfalfa (m^3^/ha).

#### 4.2.3. Data Analysis

The mean values of replicate measurements were used for each experimental treatment. To facilitate a comparison between the two growing seasons, the response parameters in the violin plots were converted to average values per cutting cycle, while other analytical graphs represented annual totals. Statistical analyses were conducted using Design-Expert software (Trial version 13, Stat-Ease Inc., Minneapolis, MN, USA), encompassing analysis of variance (ANOVA) and model quality assessments. This software was also utilized for multi-response optimization. To evaluate the significance and importance of each parameter in our regression model, we computed the t-statistic for each estimated coefficient. This involves standardizing the effect of each parameter by dividing the coefficient by its standard error [69]. Independent samples *t*-tests for the indices of the two growing seasons were conducted using SPSS 27 (SPSS Inc., Chicago, IL, USA). Graphical representations were created using Origin 2021 (Origin Lab Corporation, Northampton, MA, USA) and Design-Expert software. All statistical tests were conducted at a significance level of *α* = 0.05 unless otherwise specified. Prior to analysis, data were checked for normality using the Shapiro–Wilk test and for homogeneity of variances using Levene’s test. When necessary, data transformations were applied to meet the assumptions of parametric tests.

## 5. Conclusions

Based on the comprehensive analysis of water–fertilizer coupling in alfalfa using RSM-CCD methodology, this study systematically evaluated the contributions and interactions of multiple variables (water regulation during the branching period, water regulation during the bud formation period, nitrogen fertilizer, and phosphorus fertilizer) on alfalfa yield, water consumption, and WUE. The established regression models demonstrated a high correlation with measured values. The main conclusions are as follows:

(1) The order of factor importance affecting alfalfa yield and water consumption over two years was: water stress during the branching period > water stress during the bud formation period > phosphorus fertilizer > nitrogen fertilizer. However, nitrogen and phosphorus fertilizers were identified as crucial factors in improving water use efficiency.
(2) Both nitrogen and phosphorus fertilizers exhibited quadratic parabolic effects on the three parameters. As fertilizer application rates increased, yield and water use efficiency (WUE) initially increased and then decreased, while water consumption showed an opposite trend of first decreasing and then increasing.
(3) Under optimal RSM conditions, the appropriate combination of regulated deficit irrigation and nitrogen–phosphorus fertilization enhanced crop yield and WUE while maintaining relatively low water consumption levels.

Additionally, this study revealed the advantageous carry-over effects of regulated deficit irrigation and N-P fertilizer synergy on alfalfa production in subsequent years. These findings provide valuable theoretical guidance for efficient alfalfa production in arid regions, facilitating optimal resource allocation and improved water use efficiency, thereby promoting sustainable development of the alfalfa industry.

## Figures and Tables

**Table 1:** Measured parameters of alfalfa cultivation based on the Central Composite Design.

Response		Depended Variable	Range	Mean	Std Dev
2022	*Y*_1_ (Yield)	Hay yield (g/pot)	165.19–462.87	299.08	80.13
	*Y*_2_ (ET)	Water consumption (10^−3^·m^3^/pot)	13.93–38.87	23.77	6.42
	*Y*_3_ (WUE)	Water use efficiency (kg/m^3^)	9.04–15.94	12.72	1.84
2023	*Y*_4_ (Yield)	Hay yield (g/pot)	235.99–678.37	507.01	125.75
	*Y*_5_ (ET)	Water consumption (10^−3^·m^3^/pot)	20.33–49.53	33.21	6.89
	*Y*_6_ (WUE)	Water use efficiency (kg/m^3^)	9.47–19.37s	15.30	2.77

**Table 2:** Mathematical models for estimation the parameters of alfalfa cultivation.

Equation Number	Mathematical Models	F-Value	*p*-Value		Summary Statistics				
			(Prob > F)						
			Model	LF	*R* ^2^	*AR* ^2^	*PR* ^2^	AP	CV
(**1**)	$Y_{1}=304.28+68.22A+43.05B+9.35C+18.48D+17.82\mathrm{AB}+4.79\mathrm{AC}+5.63\mathrm{AD}+2.40\mathrm{BC}$ $\;\;\;\;\;\;\;\;\;\;\;\;\;\;\;\;\;\;\;\;\;\;\;\;\;\;\;\;\;\;\;\;\;\;\;\;\;\;+3.21\mathrm{BD}-7.16\mathrm{CD}+4.47A^{2}+7.85B^{2}-9.27C^{2}-9.56D^{2}$	42.85	<0.0001	0.6455	0.9756	0.9528	0.9001	22.6089	5.82
(**2**)	$Y_{2}=20.52+5.65A+3.36B-0.3838C-0.7413D+0.4644\mathrm{AB}+0.1144\mathrm{AC}-0.3056\mathrm{AD}+0.2081\mathrm{BC}$ $-0.2719\mathrm{BD}+1.14\mathrm{CD}+1.60A^{2}\;+0.9236B^{2}\;+0.8024C^{2}\;+0.7461D^{2}$	63.52	<0.0001	0.4425	0.9834	0.9679	0.9254	29.3266	4.84
(**3**)	$Y_{3}=14.85-0.0492A-0.0792B+0.5283C+1.06D+0.4938\mathrm{AB}-0.0025\mathrm{AC}+0.1650\mathrm{AD}-0.1000\mathrm{BC}$ $+0.1750\mathrm{BD}-0.8362\mathrm{CD}-0.7083A^{2}-0.2033B^{2}-0.8883C^{2}-0.8521D^{2}$	19.65	<0.0001	0.9937	0.9483	0.9000	0.8725	13.6607	4.58
(**4**)	$Y_{4}=558.53+119.11A+34.84B+18.80C+30.46D-24.90\mathrm{AB}+9.51\mathrm{AC}+14.13\mathrm{AD}+0.2850\mathrm{BC}$ $+3.19\mathrm{BD}-21.30\mathrm{CD}-29.45A^{2}-10.67B^{2}-11.85C^{2}-12.43D^{2}$	55.73	<0.0001	0.1610	0.9811	0.9635	0.9049	25.0582	4.74
(**5**)	$Y_{5}=30.18+6.47A+3.13B-0.1546C-1.01D-0.2544\mathrm{AB}-0.1669\mathrm{AC}+0.1631\mathrm{AD}-0.1356\mathrm{BC}$ $-0.6156\mathrm{BD}+1.48\mathrm{CD}+1.74A^{2}\;+0.6130B^{2}\;+0.7418C^{2}\;+0.6855D^{2}$	40.47	<0.0001	0.3361	0.9742	0.9501	0.8790	25.3256	4.64
(**6**)	$Y_{6}=18.53+1.03A-0.2138B+0.5177C+1.29D-0.6516\mathrm{AB}+0.3046\mathrm{AC}+0.0924\mathrm{AD}-0.00453\mathrm{BC}$ $+0.1988\mathrm{BD}-1.24\mathrm{CD}-1.85A^{2}-0.6722B^{2}-0.7693C^{2}-0.7444D^{2}$	31.33	<0.0001	0.6817	0.9669	0.9361	0.8672	19.1064	4.58

Model terms with “Prob > F” values below 0.05 are considered significant. LF represents Lack of Fit, while *R*^2^, *AR*^2^, and *PR*^2^ indicate the Coefficient of Determination, adjusted *R*^2^, and predicted *R*^2^, respectively. AP signifies the Signal-to-Noise Ratio and CV is the Coefficient of Variation, which is calculated as the ratio of the standard deviation to the mean. In the equations of the table, A denotes water regulation during the branching stage, B signifies water regulation during the budding stage, C represents nitrogen fertilization, and D indicates phosphorus fertilization.

**Table 3:** Constraints and desirability on the variables used for optimization in 2022 and 2023.

Name	Goal	Lower Limit	Upper Limit	Weight	Importance	Desirability
A: Branching stage water	is in range	70	90	1	3	1.000
B: Budding stage water	is in range	70	90	1	3	1.000
C: Nitrogenous fertilizer	is in range	70	210	1	3	1.000
D: Phosphate fertilizer	is in range	70	210	1	3	1.000
Yield in 2022	maximize	165.19	462.87	1	4	0.755
ET in 2022	minimize	13.93	38.87	1	3	0.516
WUE in 2022	maximize	9.04	15.94	1	4	0.721
Yield in 2023	maximize	235.99	678.37	1	4	0.838
ET in 2023	minimize	20.33	49.53	1	3	0.629
WUE in 2023	maximize	9.47	19.37	1	4	1.000

In the table, ET denotes water consumption and WUE refers to water use efficiency.

**Table 4:** Main physicochemical properties of the experimental soil (0–30 cm).

Property	Field Capacity (cm^3^/cm^3^)	Bulk Density (g/cm^3^)	pH	Organic Matter (g/kg)	Total N (g/kg)	Available N (mg/kg)	Available P (mg/kg)	Available K (g/kg)
Value	28.2	0.92	7.4	16.58	0.28	68.83	3.68	0.22

**Table 5:** Ranges and levels of the independent factors.

Symbols	Variables	Units	Ranges and Levels				
			−2	−1	0	1	2
A (W1)	Branching stage water	%	60	70	80	90	100
B (W2)	Budding stage water	%	60	70	80	90	100
C (N)	Nitrogenous fertilizer	kg/ha	0	70	140	210	280
D (P)	Phosphate fertilizer	kg/ha	0	70	140	210	280

**Table 6:** The experimental design matrix based on the CCD.

Runs	Space Type	Factor 1	Factor 2	Factor 3	Factor 4
		Branching Stage Water %	Budding Stage Water %	Nitrogenous Fertilizer kg/ha	Phosphate Fertilizer kg/ha
1	Axial	80	80	280	140
2	Center	80	80	140	140
3	Center	80	80	140	140
4	Factorial	90	70	70	70
5	Axial	80	100	140	140
6	Axial	80	80	140	280
7	Factorial	90	70	210	210
8	Factorial	90	90	210	70
9	Axial	80	80	0	140
10	Center	80	80	140	140
11	Factorial	90	90	70	70
12	Axial	100	80	140	140
13	Factorial	70	90	210	210
14	Factorial	90	70	210	70
15	Factorial	70	70	70	70
16	Factorial	70	70	210	210
17	Axial	80	80	140	0
18	Center	80	80	140	140
19	Axial	80	60	140	140
20	Center	80	80	140	140
21	Factorial	90	90	210	210
22	Axial	60	80	140	140
23	Factorial	70	90	70	210
24	Factorial	70	90	70	70
25	Factorial	70	70	210	70
26	Factorial	90	70	70	210
27	Center	80	80	140	140
28	Factorial	70	90	210	70
29	Factorial	90	90	70	210
30	Factorial	70	70	70	210

Notes: The irrigation levels in the table represent the upper soil moisture limits, all expressed as percentages of 85% field capacity (denoted as θ_0.85_). Fertilizer amounts indicate the total annual application. Potassium fertilizer was applied uniformly across all treatments at 210 kg/ha.

## Data Availability

All data are incorporated into the article.
